# Associations between cardiovascular-kidney-metabolic syndrome and disability in activities of daily living: a nationwide longitudinal study among the middle-aged and older adults in China

**DOI:** 10.3389/fpubh.2024.1480576

**Published:** 2025-01-15

**Authors:** Junfeng Zhang, Huijie Huang, Zhan Lin, Jingran Dong, Xiaoyan Zhang, Jing Gao, Yin Liu, Changping Li, Zhuang Cui

**Affiliations:** ^1^Department of Epidemiology and Biostatistics, School of Public Health, Tianjin Medical University, Tianjin, China; ^2^Thoracic Clinical College, Tianjin Medical University, Tianjin, China; ^3^Cardiovascular Institute, Tianjin Chest Hospital, Tianjin, China; ^4^Department of Cardiology, Tianjin Chest Hospital, Tianjin, China

**Keywords:** cardiovascular-kidney-metabolic syndrome, disability, activities of daily living, diabetes, metabolic risk factors, chronic kidney diseases, cardiovascular diseases, CHARLS

## Abstract

**Background:**

Activities in daily living (ADLs) disability triggered by aging population and chronic diseases in the middle-aged and older adults has become a major public health challenge. Cardiovascular-kidney-metabolic (CKM) syndrome, as a combination of several chronic conditions, has not yet been studied to explore its association with ADLs disability. We examined the association between CKM syndrome and ADLs disability among middle-aged and older adults in China and whether it varied by age and socioeconomic status.

**Methods:**

Participants were from China Health and Retirement Longitudinal Study (CHARLS), which conducted four waves of surveys from 2011 to 2018. CKM stage was calculated through disease and physical examination data from CHARLS database. Meanwhile, the degree of disability was assessed through the ADL scale. Generalized linear mixed model was used to perform multivariate analysis to explore the association between CKM syndrome and the risk of ADLs disability.

**Results:**

The proportion of CKM stage 0, 1, 2, and 3 among the 5,898 eligible participants (median age 60 years, 60.27% women) in 2011 were 14.70, 30.23, 41.39, and 13.68%. The risk of ADL disability was increased by 16% (odds ratio [95% confidence interval]; 1.16 [1.00–1.33]) and 33% (1.33 [1.12–1.58]) in CKM stages 2 and 3 compared with stage 0. In addition, there was a greater risk of BADL disability in 75+ age group compared to other age groups, but no significant association with IADL disability. In the subgroup aged 75+, the risk of BADL disability was increased for CKM stage 2/3 (1.48 [1.01–2.18]/1.67 [1.06–2.64]) compared with stage 0. Only in the lowest quartile of socioeconomic status group CKM stage rise was strongly associated with the risk of disability. The risk of ADL disability was greater for CKM stage 2/3 (1.45 [1.15–1.83]/1.48 [1.11–1.98]) compared to CKM stage 0 in the lowest economic status quartile.

**Conclusion:**

For middle-aged and older adults in China, CKM syndrome is a key risk factor for ADLs disability. Therefore, effective measures should be taken to manage CKM stage at the lowest possible level, especially in older and economically disadvantaged populations.

## Introduction

Diabetes, chronic kidney disease (CKD) and cardiovascular disease (CVD) are three major challenges in global health, and their high and increasing prevalence in middle-aged and older adults not only affects the quality of life of individuals, but also poses a significant burden on global health and the economy (1–3). Diabetes affects approximately 9.3% of the global population and more than 460 million people. In China, the number of diabetes cases has surged and is expected to reach 140 million by 2030 (4). CKD affects about 5–7% of the global population, especially in developing countries and among vulnerable groups (5), a trend mirrored in China, where cases of CKD are on the rise, especially among people with diabetes and hypertension (6). CVD remains the leading cause of death globally, claiming 17.7 million lives annually and accounting for 31% of global deaths, with China bearing a heavy burden, especially among the older adults (5). In view of this, the Chinese Government has proposed a series of measures to curb the high prevalence of these chronic conditions. For example, the Healthy China Initiative (7) has developed programs to prevent and treat CVD and diabetes, and the Healthy Poverty Alleviation Project (8) provides chronic disease screening and treatment services targeting impoverished areas. However, due to population aging and the prevalence of risk factors for chronic conditions, the burden of chronic conditions in China continues to increase and is likely to continue to grow (9). It is considered that these conditions frequently coexist (10). To better understand the complex interactions between metabolic risk factors (e.g., obesity and diabetes mellitus), CKD, and the cardiovascular system, the American Heart Association introduced the concept of CKM syndrome in a 2023 presidential advisory note (11). CKM syndrome is defined as a spectrum of disease states associated with dysfunction or excessive obesity. CKM health is the clinical manifestation of pathophysiologic interactions between metabolic risk factors, CKD, and cardiovascular system. Adverse CKM health leads to serious clinical consequences, including premature mortality, excess morbidity, and multiorgan diseases (11).

Disability is generally recognized as difficulties in performing activities necessary for daily living (12). The most common problem affecting the health and quality of life of older adults is functional disability. Functional disability in older adults is defined as acquired difficulties in accomplishing basic daily tasks or more complex tasks required for independent living (13). The most widely used measures of functional disability are basic activities of daily living (BADL), which are daily activities that people perform without assistance such as personal care, hygiene, mobility, and eating, and instrumental activities of daily living (IADL), which are higher level activities associated with independent living in the community such as cooking, shopping, housekeeping, and money management (14, 15). According to the World Health Survey across 59 countries, the average prevalence of severe ADLs disability among adults aged 60 and above is 38.1%, varying from 29.5% in high-income countries to 43.4% in low-income countries (16). ADLs disability poses significant challenges to global health and social care systems, increasing the burden on healthcare services and family caregivers (17). It is associated with poorer quality of life (18) and higher mortality rates (19).

Both CKM syndrome and ADLs disability are common in the older adults population (16, 20), and there is no research evidence to support an association between them. CKM syndrome is a multi-system chronic condition involving the heart, kidneys, and metabolic system. In previous studies, chronic conditions affect patients’ physical, psychological, and social functioning, not just their physical health (21). Established theories such as the biopsychosocial model suggest that the functional disability of patients with chronic diseases does not only originate from the physical disease, but is also influenced by the psychological state and social environment (22). This gives us a new clue that CKM syndrome may trigger functional disability by affecting patients’ physical health, psychological state and social functioning. For patients with CKM syndrome, the multiple physical impairments of CKM syndrome, such as CVD (23, 24), CKD (25), metabolic syndrome, and obesity (26), may lead to a decrease in the patient’s ability to perform daily activities by directly affecting physical function. In addition, diabetes (27) and CVD (28) are often accompanied by mental health problems such as depression and anxiety, and these physical and psychological factors can further limit the patient’s social activities and ability to work (29–31), increasing functional disability in life. However, few prospective studies have explored the mutual relationship between CKM syndrome and ADLs disability and how this relationship varies across age and economic status groups.

To enhance our understanding of this relationship, our study utilized data from the CHARLS to estimate the association between CKM syndrome and ADLs disability from 2011 to 2018, and explored differences by age group and socioeconomic status.

## Methods

### Study design and data sources

This study is based on the CHARLS, a national longitudinal survey of individuals aged 45 years and older. CHARLS has conducted five waves of surveys to date, with the baseline beginning in 2011 (Wave 1). A total of 17,705 participants from 450 villages or communities across 28 provinces were recruited using the probability proportionate to size (PPS) Sampling technique. These participants were then followed up in 2013, 2015, 2018, and 2020. Information on participants’ sociodemographics, health status, physical functioning, family characteristics, and lifestyle was collected through one-on-one interviews and structured questionnaires. The overall response rate for the first wave of CHARLS was 80.5% (32). The data included individual weighting variables to ensure that the survey sample was nationally representative. CHARLS received ethical approval from the Ethics Review Committee of Peking University under the approval number IRB00001052-11015, and each participant signed a written informed consent form.

**Figure 1:**
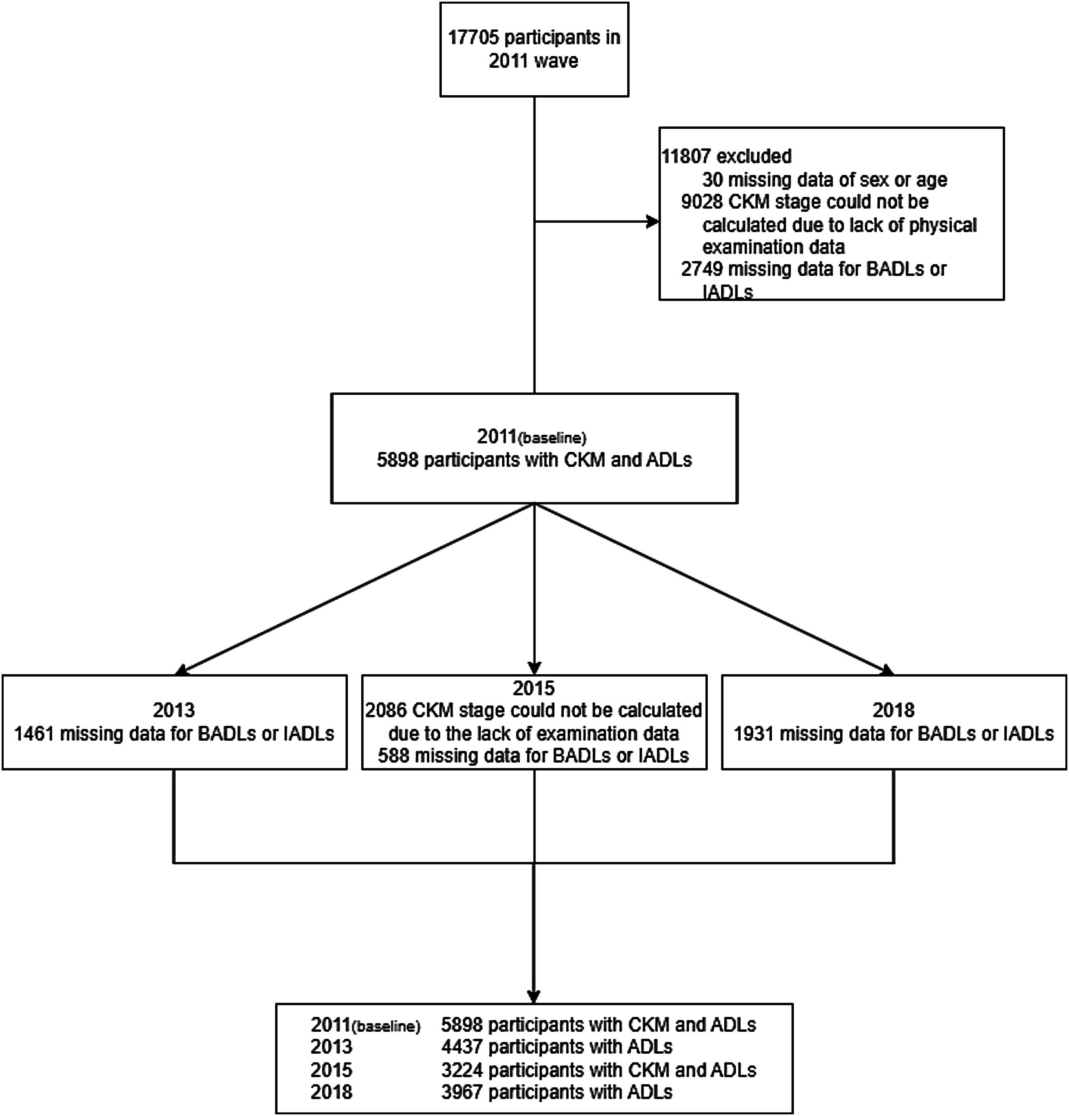
Flowchart of the selection of the study population.

In our population-based panel data analysis, we utilized data from four waves of CHARLS in 2011, 2013, 2015, and 2018. In this study, we included observations from participants with no missing CKM and ADLs disability data for each wave. We excluded participants with missing age-sex information, missing individual weights, and those without baseline observations. Figure 1 illustrates our study population screening process.

### Assessment of CKM syndrome

**Figure 2:**
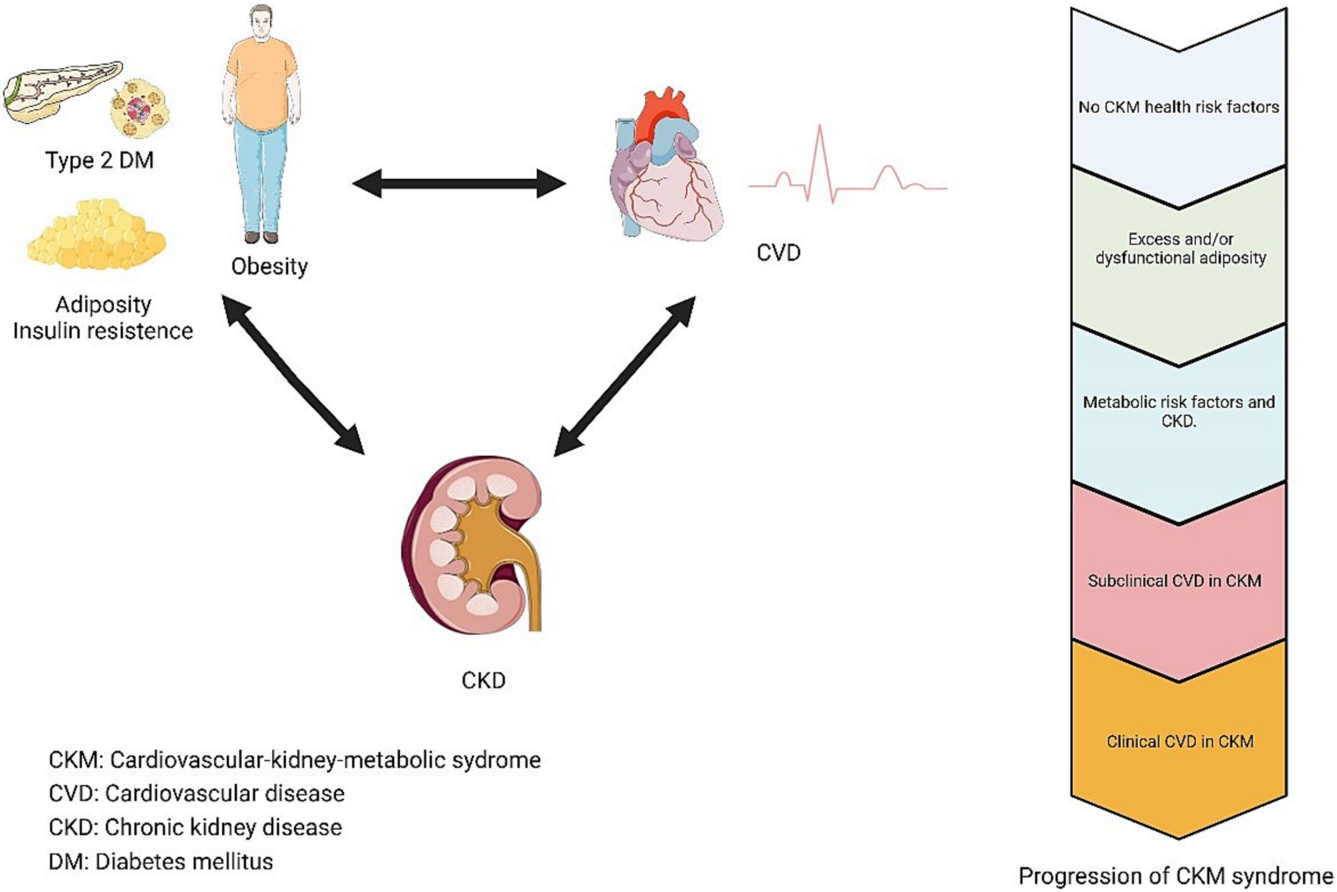
Conceptual diagram for CKM syndrome.

According to the presidential advisory note issued by the American Heart Association (AHA) in early October 2023 (11), CKM syndrome is categorized into four different stages, ranging from stage 0 to stage 4 (Figure 2). We adapted the evaluation criteria for CKM to the Chinese population, and the following definitions of each CKM stage were presented:

Stage 0: No CKM health risk factors. Individuals without overweight/obesity, metabolic risk factors (hypertension, hypertriglyceridemia, metabolic syndrome, diabetes), CKD, or subclinical/clinical CVD.

Stage 1: Excess and/or dysfunctional adiposity. Individuals with overweight/obesity, abdominal obesity, or dysfunctional adipose tissue without the presence of other metabolic risk factors or CKD. BMI ≥ 23 kg/m^2^ waist circumference ≥ 80 cm in women and ≥ 90 cm in men. Fasting blood glucose ≥100–124 mg/dL or HbA1c between 5.7 and 6.4%.

Stage 2: Metabolic risk factors and CKD. Individuals with metabolic risk factors (hypertriglyceridemia, hypertension, diabetes mellitus, metabolic syndrome) or CKD.

Stage 3: Subclinical CVD in CKM: Subclinical Atherosclerotic cardiovascular disease or subclinical heart failure among individuals with excess/dysfunctional adiposity, other metabolic risk factors, or CKD. Or at equivalent risk (predicted high-risk cardiovascular disease or very high-risk chronic kidney disease).

Stage 4: Clinical CVD in CKM: Clinical CVD (coronary heart disease, heart failure, stroke, peripheral artery disease, Atrial fibrillation) among individuals with excess/dysfunctional adiposity, other metabolic risk factors, or CKD. Of these, stage 4a without renal failure and stage 4b with renal failure.

It was difficult to obtain the prevalence of subclinical CVD in participants from the CHARLS database. Therefore, to more accurately characterize CKM stages with physical examination data from the database, we combined stages 3 and 4 and referred to them as CKM stage 3 in this study.

### Assessment of ADLs disability

The ADLs disability was measured using the BADL and IADL scales. BADL reflected six items measuring dressing, bathing, eating, getting up, toileting, and continence. IADL consisted of five items that measure housework, cooking, shopping, taking medications, and handling finances. Each item consisted of four response options (1. Have no difficulty; 2. Have difficulty but can still do it; 3. Have difficulty and need help; 4. Cannot do it). Based on previous studies, the outcomes were dichotomous, with respondents assigned a value of 1 if they had difficulty with any of the activities or were unable to perform the activity (options 2, 3, or 4), and a value of 0 if they reported no difficulty (option 1) in completing the activity (17, 33).

### Covariates

The covariates of interest covered social demographic characteristics, economic status, lifestyle and health conditions. Social demographic characteristics included age, sex (male, female), educational level (primary school and below, middle school, high school and above), and marital status (married or partnered, other). We categorized the economic status of the population into four levels based on annual consumption quartiles. Lifestyle features included self-reported current smoking status (yes or no) and current alcohol consumption status (yes or no). For health status, participants’ chronic disease status was assessed by self-report and classified according to the number of chronic diseases other than CKM (0, 1, 2, ≥3). Participants’ self-assessed health status was divided into three categories (good/very good, fair, and poor/very poor).

### Statistical analysis

In this study, all descriptive analyses were weighted to account for the complexity, the multistage design of the study, and non-response in the CHARLS data. The distribution of categorical variables was described by n (%), and the chi-square test was employed on the weighted data to analyze whether there existed a statistical correlation between exposure factors and CKM risk.

We used generalized linear mixed model to estimate the association between CKM stages and ADLs disability. The link function was set to logit. We considered the baseline CKM stage as the stage for all follow-up time points. Adjusted odds ratios (ORs) were reported based on age, sex, marital status, education level, economic status, presence of other chronic diseases, self-assessed health, smoking, and alcohol consumption, with a 95% confidence interval. We also assessed variable covariance using the variance inflation factor (VIF). Generally, VIF values <10 is considered acceptable. Generalized linear mixed model was used to evaluate the association between CKM stages and ADLs disability in 2011 and 2015. In the generalized linear mixed model for 2011 and 2015, CKM stage and functional disability status for the respective years were used. Detailed health examination data for both years allowed us to calculate CKM stage for the respective time periods. To explore differential effects in the population, we conducted stratified analyses by age and socioeconomic status.

We conducted the following sensitivity analyses: (1) We transformed BADL, IADL, and ADL disabilities from dichotomous categories into scores and explored the association between CKM stage and scores for ADL, BADL, and ADL with linear mixed-effects model. For all questions in the BADL and IADL scales, the 4 options (1. Have no difficulty; 2. Have difficulty but can still do it; 3. Have difficulty and need help; 4. Cannot do it) were assigned values of 0, 0.33, 0.66, and 1, respectively. (2) The multiple imputation by chained equations method was applied to impute missing covariates in our study, generating five imputed datasets. We combined the estimated values from the generalized linear mixed models for each dataset from the five imputed datasets.

All analyses were conducted using statistical software R 4.4.0, and two-tailed *p*-values less than 0.05 were considered statistically significant.

## Results

### Baseline characteristics of study participants

Of the 17,705 participants in charls2011, 5,898 were eligible for inclusion in our analyses. The sociodemographic characteristics of qualified participants are shown in Table 1. Median age of the participants in 2011 was 60 years (IQR 54–68). Among the participants, 2,343 (39.73%) were male, and 3,555 (60.27%) were female. 4,545 participants (77.06%) had an educational level of primary school or below. 5,061 participants (85.81%) were married or partnered. 3,604 (61.11%) had at least 1 chronic disease. 3,699 (62.72%) perceived their health as poor or very poor.1552 (26.31%) were current smokers and 1,642 (27.84%) currently drank alcohol. The percentage of CKM stages 0, 1, 2, and 3 among eligible participants in 2011 was 14.70, 30.23, 41.39, and 13.68%, respectively. The distribution of CKM stages varied across different sex, age, marital status, number of other chronic diseases, and smoking and alcohol consumption groups (all with *p* < 0.001). The females, those aged 65–74, unmarried, having three or more chronic diseases and with poorer self-rated health were more likely than their counterparts to have more severe CKM syndrome. The overall prevalence of ADL, BADL, and IADL disabilities were 38.10, 24.13, and 27.96%, respectively. In addition, the more severe CKM syndrome had greater rates of ADLs disability.

**Table 1:** Distribution of characteristics by CKM stage from CHARLS (2011).

	All (*n* = 5,898)	CKM stage 0 (*n* = 867, 14.70%)			CKM stage 1 (*n* = 1783, 30.23%)			CKM stage 2 (*n* = 2,441, 41.39%)			CKM stage 3 (*n* = 807, 13.68%)			*p*-value
	*n* (%)	*n*	Unweighted%	Weighted%	*n*	Unweighted%	Weighted%	*n*	Unweighted%	Weighted%	*n*	Unweighted%	Weighted%	
Sex														<0.001
Male	2,343(39.73)	501	21.38	22.47	803	34.27	33.66	742	31.67	31.61	297	12.68	12.26	
Female	3,555(60.27)	366	10.30	10.78	980	27.57	27.63	1,699	47.79	47.96	510	14.35	13.63	
Age														<0.001
45–54	1,649(27.96)	249	15.10	17.01	595	36.08	36.34	650	39.42	38.45	155	9.40	8.21	
55–64	2,264(38.39)	319	14.09	14.61	662	29.24	28.75	943	41.65	41.89	340	15.02	14.75	
65–74	1,378(23.36)	198	14.37	14.21	356	25.83	25.05	600	43.54	43.79	224	16.26	16.94	
≥75	607(10.29)	101	16.64	16.10	170	28.01	28.30	248	40.86	43.23	88	14.50	12.37	
Education level														0.16
Primary school and below	4,545(77.06)	682	15.01	15.65	1,389	30.56	30.29	1881	41.39	41.30	593	13.05	12.76	
Secondary school	1,304(22.11)	181	13.88	14.69	382	29.29	29.12	541	41.49	42.42	200	15.34	13.77	
Collage and above	49(0.83)	4	8.16	8.46	12	24.49	26.11	19	38.78	36.63	14	28.57	28.81	
Marital status														<0.001
Unmarried	837(14.19)	105	12.54	11.22	224	26.76	27.41	361	43.13	43.94	147	17.56	17.42	
Married or have a partner	5,061(85.81)	762	15.06	16.21	1,559	30.80	30.52	2080	41.10	41.03	660	13.04	12.23	
Socioeconomic Status														0.47
1	1,522(25.81)	211	13.86	14.61	467	30.68	30.43	649	42.64	42.90	195	12.81	12.07	
2	1,567(26.57)	255	16.27	17.10	485	30.95	30.89	613	39.12	39.29	214	13.66	12.72	
3	1,423(24.13)	213	14.97	14.95	433	30.43	30.25	584	41.04	41.60	193	13.56	13.20	
4	1,367(23.18)	185	13.53	14.83	394	28.82	28.28	585	42.79	42.41	203	14.85	14.48	
Combined with other chronic diseases														<0.001
0	2,294(38.89)	330	14.39	15.16	688	29.99	30.35	1,027	44.77	44.57	249	10.85	9.92	
1	2,122(35.98)	326	15.36	15.97	649	30.58	30.07	888	41.85	42.64	259	12.21	11.32	
2	1,082(18.35)	155	14.33	14.92	340	31.42	31.28	401	37.06	36.53	186	17.19	17.27	
>=3	400(6.78)	56	14.00	14.87	106	26.50	24.14	125	31.25	30.65	113	28.25	30.34	
Self-assessed health														<0.001
Good/very good	519(8.80)	94	18.11	18.83	174	33.53	34.41	225	43.35	41.88	26	5.01	4.87	
Fair	1,679(28.47)	248	14.77	16.05	548	32.64	32.50	723	43.06	42.62	160	9.53	8.83	
Poor/very poor	3,699(62.72)	525	14.19	14.58	1,061	28.68	28.23	1,492	40.34	40.94	621	16.79	16.24	
Current smoking														<0.001
No	4,346(73.69)	528	12.15	13.05	1,224	28.16	28.01	1967	45.26	45.20	627	14.43	13.73	
Yes	1,552(26.31)	339	21.84	22.19	559	36.02	35.82	474	30.54	30.77	180	11.60	11.23	
Current drinking														<0.001
No	4,256(72.16)	552	12.97	13.33	1,187	27.89	27.98	1874	44.03	44.46	643	15.11	14.23	
Yes	1,642(27.84)	315	19.18	20.76	596	36.30	35.30	567	34.53	33.83	164	9.99	10.12	
ADL														<0.001
No	3,651(61.90)	562	64.82	64.30	1,156	64.83	65.83	1,500	61.45	61.19	433	53.66	51.25	
Yes	2,247(38.10)	305	35.18	35.70	627	35.17	34.17	941	38.55	38.81	374	46.34	48.75	
BADL														<0.001
No	4,475(75.87)	658	75.89	75.40	1,400	78.52	79.40	1852	75.87	76.03	565	70.01	70.26	
Yes	1,423(24.13)	209	24.11	24.60	383	21.48	20.60	589	24.13	23.97	242	29.99	29.74	
IADL														<0.001
No	4,249(72.04)	653	75.32	74.86	1,334	74.82	75.27	1747	71.57	70.84	515	63.82	60.77	
Yes	1,649(27.96)	214	24.68	25.14	449	25.18	24.73	694	28.43	29.16	292	36.18	39.23	

BADL, basic activities of daily living; IADL, instrumental activities of daily living; ADL, activities of daily living; CKM: cardiovascular-kidney-metabolic syndrome.

Tables 2–4 demonstrate the distribution of different types of ADLs disability in the population. Different BADL, IADL, and ADL disabilities were distributed differently across CKM stages, age, education level, marital status, number of chronic diseases, and self-rated health populations. BADL, IADL, and ADL disabilities were associated with higher CKM stages, older age groups, lower education levels, being unmarried, more comorbid chronic diseases, and poorer self-rated health (all with *p* < 0.001).

**Table 2:** Distribution of characteristics by BADL disability from CHARLS (2011).

	All *N* = 5,898	BADL				
		Unweighted		Weighted		
		No [*n*(%)] *n* = 4,475	Yes [*n*(%)] *n* = 1,423	No (%)	Yes (%)	*P*-value
CKM stage						<0.001
0	867	658 (75.9)	209 (24.1)	75.4	24.6	
1	1783	1,400 (78.5)	383 (21.5)	79.4	20.6	
2	2,441	1852 (75.9)	589.00(24.1)	76.0	24.0	
3	807	565 (70.0)	242 (30.0)	70.3	29.7	
Sex						0.120
Male	2,343	1765 (75.3)	578 (24.7)	75.0	25.0	
Female	3,555	2,710 (76.2)	845 (23.8)	77.0	23.0	
Age						<0.001
45–54	1,649	1,405 (85.2)	244 (14.8)	85.4	14.6	
55–64	2,264	1715 (75.8)	549 (24.2)	76.2	23.8	
65–74	1,378	963 (69.9)	415 (30.1)	71.4	28.6	
≥75	607	392 (64.6)	215 (35.4)	63.9	36.1	
Education level						<0.001
Primary school and below	4,545	3,347 (73.6)	1,198 (26.4)	73.9	26.1	
Secondary school	1,304	1,089 (83.5)	215 (16.5)	84.1	15.9	
Collage and above	49	39 (79.6)	10 (20.4)	79.1	20.9	
Marital status						<0.001
Unmarried	837	589 (70.4)	248 (29.6)	71.0	29.0	
Married or have a partner	5,061	3,886 (76.8)	1,175 (23.2)	77.2	22.8	
Socioeconomic Status						<0.001
1	1,522	1,094 (71.9)	428 (28.1)	72.5	27.5	
2	1,567	1,203 (76.8)	364 (23.2)	77.0	23.0	
3	1,423	1,074 (75.5)	349 (24.5)	75.0	25.0	
4	1,367	1,089 (79.7)	278 (20.3)	80.1	19.9	
Combined with other chronic diseases						<0.001
0	2,294	1919 (83.7)	375 (16.3)	84.0	16.0	
1	2,122	1,582 (74.6)	540 (25.4)	74.5	25.5	
2	1,082	723 (66.8)	359 (33.2)	67.1	32.9	
≥3	400	251 (62.8)	149 (37.2)	63.6	36.4	
Self-assessed health						<0.001
Good/very good	519	454 (87.5)	65 (12.5)	86.3	13.7	
Fair	1,679	1,385 (82.5)	294 (17.5)	82.7	17.3	
Poor/very poor	3,699	2,635 (71.2)	1,064 (28.8)	71.8	28.2	
Current smoking						0.700
No	4,346	3,284 (75.6)	1,062 (24.4)	76.1	23.9	
Yes	1,552	1,191 (76.7)	361 (23.3)	76.6	23.4	
Current drinking						0.420
No	4,256	3,218 (75.6)	1,038 (24.4)	75.9	24.1	
Yes	1,642	1,257 (76.6)	385 (23.4)	77.0	23.0	

BADL, basic activities of daily living; CKM, cardiovascular-kidney-metabolic syndrome.

**Table 3:** Distribution of characteristics by IADL disability from CHARLS (2011).

	All *N* = 5,898	IADL				
		Unweighted		Weighted		
		No [*n*(%)] *n* = 4,475	Yes [*n*(%)] *n* = 1,423	No (%)	Yes (%)	*P*-value
CKM stage						<0.001
0	867	653 (75.3)	214 (24.7)	74.9	25.1	
1	1783	1,334 (74.8)	449 (25.2)	75.3	24.7	
2	2,441	1747 (71.6)	694 (28.4)	70.8	29.2	
3	807	515 (63.8)	292 (36.2)	60.8	39.2	
Sex						<0.001
Male	2,343	1766 (75.4)	577 (24.6)	75.0	25.0	
Female	3,555	2,483 (69.9)	1,072 (30.1)	69.2	30.8	
Age						<0.001
45–54	164	1,334 (80.9)	315 (19.1)	81.7	18.3	
55–64	2,264	1,672 (73.9)	592 (26.1)	73.9	26.1	
65–74	1,378	904 (65.6)	474 (34.4)	65.7	34.3	
≥75	607	339 (55.9)	268 (44.1)	51.6	48.4	
Education level						<0.001
Primary school and below	4,545	3,126 (68.8)	1,419 (31.2)	68.0	32.0	
Secondary school	1,304	1,080 (82.8)	224 (17.2)	83.2	16.8	
Collage and above	49	43 (87.8)	6 (12.2)	87.5	12.5	
Marital status						<0.001
Unmarried	837	529 (63.2)	308 (36.8)	59.5	40.5	
Married or have a partner	5,061	3,720 (73.5)	1,341 (26.5)	73.8	26.2	
Socioeconomic Status						0.036
1	1,522	1,047 (68.8)	475 (31.2)	68.4	31.6	
2	1,567	1,148 (73.3)	419 (26.7)	72.9	27.1	
3	1,423	1,025 (72.0)	398 (28.0)	70.6	29.4	
4	1,367	1,014 (74.2)	353 (25.8)	73.7	26.3	
Combined with other chronic diseases						<0.001
0	2,294	1809 (78.9)	485 (21.1)	79.2	20.8	
1	2,122	1,510 (71.2)	612 (28.8)	69.9	30.1	
2	1,082	705 (65.2)	377 (34.8)	64.4	35.6	
≥3	400	225 (56.3)	175 (43.7)	53.1	46.9	
Self-assessed health						<0.001
Good/very good	519	431 (83.0)	88 (17.0)	81.7	18.3	
Fair	1,679	1,347 (80.2)	332 (19.8)	79.5	20.5	
Poor/very poor	3,699	2,471 (66.8)	1,228 (33.2)	66.4	33.6	
Current smoking						<0.001
No	4,346	3,080 (70.9)	1,266 (29.1)	70.1	29.9	
Yes	1,552	1,169 (75.3)	383 (24.7)	75.4	24.6	
Current drinking						<0.001
No	4,256	3,003 (70.6)	1,253 (29.4)	70.0	30.0	
Yes	1,642	1,246 (75.9)	396 (24.1)	75.4	24.6	

IADL, instrumental activities of daily living; CKM, cardiovascular-kidney-metabolic syndrome.

**Table 4:** Distribution of characteristics by ADL disability from CHARLS (2011).

	All *N* = 5,898	ADL				
		Unweighted		Weighted		
		No [*n*(%)] *n* = 4,475	Yes [*n*(%)] *n* = 1,423	No (%)	Yes (%)	*P*-value
CKM stage						<0.001
0	867	562 (64.8)	305 (35.2)	64.3	35.7	
1	1783	1,156 (64.8)	627 (35.2)	65.8	34.2	
2	2,441	1,500 (61.5)	941 (38.5)	61.2	38.8	
3	807	433 (53.7)	374 (46.3)	51.3	48.7	
Sex						0.077
Male	2,343	1,488 (63.5)	855 (36.5)	63.4	36.6	
Female	3,555	2,163 (60.8)	1,392 (39.2)	60.7	39.3	
Age						<0.001
45–54	1,649	1,218 (73.9)	431 (26.1)	74.7	25.3	
55–64	2,264	1,414 (62.5)	850 (37.5)	62.9	37.1	
65–74	1,378	743 (53.9)	635 (46.1)	54.3	45.7	
≥75	607	276 (45.5)	331 (54.5)	42.5	57.5	
Education level						<0.001
Primary school and below	4,545	2,647 (58.2)	1898 (41.8)	57.9	42.1	
Secondary school	1,304	969 (74.3)	335 (25.7)	75.1	24.9	
Collage and above	49	35 (71.4)	14 (28.6)	70.7	29.3	
Marital status						<0.001
Unmarried	837	444 (53.1)	393 (46.9)	51.0	49.0	
Married or have a partner	5,061	3,207 (63.4)	1854 (36.6)	63.9	36.1	
Socioeconomic Status						0.001
1	1,522	870 (57.2)	652 (42.8)	57.3	42.7	
2	1,567	992 (63.3)	575 (36.7)	63.4	36.6	
3	1,423	881 (61.9)	542 (38.1)	60.9	39.1	
4	1,367	895 (65.5)	472 (34.5)	65.3	34.7	
Combined with other chronic diseases						<0.001
0	2,294	1,633 (71.2)	661 (28.8)	71.8	28.2	
1	2,122	1,282 (60.4)	840 (39.6)	59.3	40.7	
2	1,082	559 (51.7)	523 (48.3)	51.3	48.7	
≥3	400	177 (44.3)	223 (55.7)	42.7	57.3	
Self-assessed health						<0.001
Good/very good	519	394 (75.9)	125 (24.1)	74.0	26.0	
Fair	1,679	1,199 (71.4)	480 (28.6)	71.6	28.4	
Poor/very poor	3,699	2058 (55.6)	1,641 (44.4)	55.5	44.5	
Current smoking						0.051
No	4,346	2,654 (61.1)	1,692 (38.9)	61.0	39.0	
Yes	1,552	997 (64.2)	555 (35.8)	64.1	35.9	
Current drinking						0.058
No	4,256	2,603 (61.2)	1,653 (38.8)	60.9	39.1	
Yes	1,642	1,048 (63.8)	594 (36.2)	64.0	36.0	

ADL, activities of daily living; CKM, cardiovascular-kidney-metabolic syndrome.

### The association between CKM and ADLs disability

In the overall sample, participants in CKM stages 2 and 3 had 22% (aOR = 1.22, 95% CI: 1.03–1.45) and 42% (aOR = 1.42, 95CI: 1.16–1.74) greater risks of BADL disability than those in CKM stage 0, respectively. CKM stage 3 was associated with a greater risk of IADL disability compared to CKM stage 0 (aOR = 1.31, 95% CI: 1.09–1.57). The risk of ADL disability was 16% greater for CKM stage 2 (aOR = 1.16, 95% CI: 1.00–1.33) and 33% greater for CKM stage 3 (aOR = 1.33, 95% CI: 1.12–1.58) compared to CKM stage 0 (Table 5). The VIF values of all variables in the models were <2, indicating that there was no multicollinearity between the variables. The association between CKM stage and ADLs disability in the 2011 and 2015 waves was generally consistent with our main findings (Table 6).

**Table 5:** Association between the stage of CKM and ADLs disability in China, 2011–2018.

Stage of CKM	BADL disability		IADL disability		ADL disability	
	OR (95% CI)*	*P*-value	OR (95% CI)*	*P*-value	OR (95% CI)*	*P*-value
0	ref	–	ref	–	ref	–
1	1.05(0.88–1.25)	0.576	1.01(0.86–1.18)	0.911	1.07(0.92–1.23)	0.401
2	**1.22(1.03–1.45)**	0.019	1.08(0.93–1.25)	0.326	**1.16(1.00–1.33)**	0.046
3	**1.42(1.16–1.74)**	<0.001	**1.31(1.09–1.57)**	0.004	**1.33(1.12–1.58)**	0.001

OR, odds ratio; CI, confidence interval; BADL, basic activities of daily living; IADL, instrumental activities of daily living; ADL, activities of daily living; CKM, cardiovascular-kidney-metabolic syndrome.

*All estimates were adjusted for age, sex, marital status, education, number of other non-communicable diseases, self-reported health, smoking, drinking and socioeconomic status.

Bold values indicate statistical significance at the 0.05 level.

**Table 6:** Association between the stage of CKM and ADLs disability in China, 2011 and 2015.

Stage of CKM	BADL disability		IADL disability		ADL disability	
	OR (95% CI)*	*P*-value	OR (95% CI)*	*P*-value	OR (95% CI)*	*P*-value
0	ref	–	ref	–	ref	–
1	1.24(1.00–1.55)	0.050	1.12(0.92–1.37)	0.261	**1.33(1.10–1.61)**	0.004
2	**1.24(1.01–1.53)**	0.038	**1.21(1.00–1.46)**	0.047	**1.33(1.11–1.59)**	0.002
3	**1.32(1.03–1.70)**	0.030	**1.38(1.10–1.74)**	0.006	**1.39(1.11–1.74)**	0.004

OR, odds ratio; CI, confidence interval; BADL, basic activities of daily living; IADL, instrumental activities of daily living; ADL, activities of daily living; CKM, cardiovascular-kidney-metabolic syndrome.

*All estimates were adjusted for age, sex, marital status, education, number of other non-communicable diseases, self-reported health, smoking, drinking and socioeconomic status.

Bold values indicate statistical significance at the 0.05 level.

### Stratified analysis

**Figure 3:**
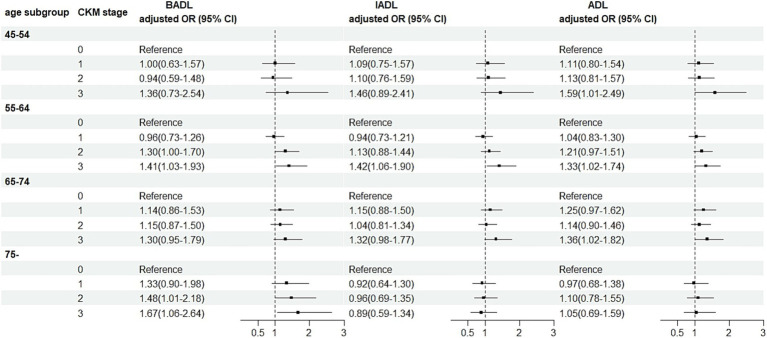
Association between CKM and ADLs disability by age groups. OR, odds ratio; BADL, basic activities of daily living; IADL, instrumental activities of daily living; ADL, activities of daily living; CKM, cardiovascular-kidney-metabolic syndrome. All estimates were adjusted for age, sex, marital status, education, number of other non-communicable diseases, self-reported health, smoking, drinking and socioeconomic status.

Age-stratified analyses showed that, overall, differences in associations between CKM stage and BADL, IADL, and ADL disabilities were similar across age subgroups. Across age subgroups, rising CKM stage was associated with an increased risk of disability in BADL, particularly evident in the 75 years and older subgroup. In the 75 years and older age subgroup, the risk of CKM stage 2 and stage 3 BADL was increased by 48% (aOR = 1.48, 95% CI: 1.01–2.18) and 67% (aOR = 1.67, 95% CI: 1.06–2.64), respectively, compared with stage 0. Within each age subgroup for IADL and ADL disabilities, higher CKM stage is associated with a greater risk of disability compared to stage 0, except in the 75 years and older subgroup (Figure 3).

**Figure 4:**
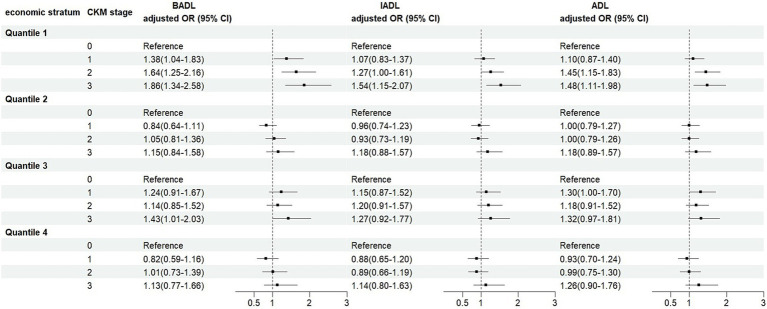
Association between CKM and ADLs disability by socioeconomic strata. OR, odds ratio; BADL, basic activities of daily living; IADL, instrumental activities of daily living; ADL, activities of daily living; CKM, cardiovascular-kidney-metabolic syndrome. All estimates were adjusted for age, sex, marital status, education, number of other non-communicable diseases, self-reported health, smoking, drinking and socioeconomic status.

Stratified analyses by socioeconomic status showed that, overall, rising CKM stage was associated with increased risk of ADLs disability across economic status quartiles. This risk was highest in the lowest economic status quartile and lowest in the highest economic status quartile (Figure 4).

### Sensitivity analysis

Our sensitivity analysis modified the calculation method for ADLs disabilities from binary classification to scoring, which was consistent with our main finding that the risk of ADLs disability increased as CKM stage progressed (Table 7). The analysis after multiple imputations also yielded similar results (Table 8).

**Table 7:** Association between the stage of CKM and ADLs score in China, 2011–2018.

Stage of CKM	BADL disability		IADL disability		ADL disability	
	Estimate *β* (95% CI)*	*p*-value	Estimate *β* (95% CI)*	*P*-value	Estimate *β* (95% CI)*	*P*-value
0	ref	–	ref	–	ref	–
1	0.02(−0.02,0.05)	0.318	0 (−0.05,0.06)	0.972	0.02(−0.06,0.10)	0.665
2	**0.05(0.02,0.09)**	0.002	**0.06(0.01,0.11)**	0.030	**0.11(0.03,0.20)**	0.005
3	**0.08(0.03,0.12)**	<0.001	**0.15(0.08,0.21)**	<0.001	**0.23(0.13,0.33)**	<0.001

CI, confidence interval; BADL, basic activities of daily living; IADL, instrumental activities of daily living; ADL, activities of daily living; CKM, cardiovascular-kidney-metabolic syndrome.

*All estimates were adjusted for age, sex, marital status, education, number of other non-communicable diseases, self-reported health, smoking, drinking and socioeconomic status.

Bold values indicate statistical significance at the 0.05 level.

**Table 8:** Association between the stage of CKM and ADLs disability in China after multiple imputation, 2011–2018.

Stage of CKM	BADL disability		IADL disability		ADL disability	
	OR (95% CI)*	*P*-value	OR (95% CI)*	*P*-value	OR (95% CI)*	*P*-value
0	ref	–	ref	–	ref	–
1	1.07(0.90–1.26)	0.437	1.00(0.86–1.17)	0.970	1.08(0.93–1.24)	0.312
2	**1.24(1.06–1.46)**	0.008	1.06(0.92–1.23)	0.418	**1.18(1.03–1.36)**	0.015
3	**1.39(1.15–1.68)**	<0.001	**1.28(1.07–1.52)**	0.006	**1.33(1.12–1.57)**	<0.001

OR, odds ratio; CI, confidence interval; BADL, basic activities of daily living; IADL, instrumental activities of daily living; ADL, activities of daily living; CKM, cardiovascular-kidney-metabolic syndrome.

*All estimates were adjusted for age, sex, marital status, education, number of other non-communicable diseases, self-reported health, smoking, drinking and socioeconomic status.

Bold values indicate statistical significance at the 0.05 level.

## Discussion

To the best of our knowledge, this study is the first to investigate the impact of CKM syndrome on ADLs disability among Chinese middle-aged and older adults. Although earlier studies have examined the impact of CVD, CKD, diabetes, and multimorbidity on ADLs disability, the impact of metabolic risk factors, CKD, and the cardiovascular system as a combined disease state on ADLs disability has not been explicitly discussed.

According to our study, there was a statistically significant association between CKM syndrome and increased risk of ADLs disability. This association persisted after adjustment for other factors in the model. In the present study, the association between rising CKM stage and ADLs disability was similar in every age group; that is, as CKM stages increased, so did the risk of ADLs disability. Besides, there was a greater risk of BADL disability in the 75- and -over age group compared with the other age groups, but there was no similar association with IADL disability. This indicated that the decline in BADL ability is more pronounced in the older population, while the decline in IADL ability may be influenced by other factors; age masked the impact of CKM stage progression on IADL impairment, which may suggest a cohort effect, indicating that people aged 75 and above have a greater lack of community-based independent living capacity than younger people. Among different socioeconomic statuses, only the group in the lowest quartile of socioeconomic status showed a strong association between rising CKM stage and risk of ADLs disability, suggesting that socioeconomic status may be an important factor influencing disability risk. This may be due to low-income groups facing limitations in accessing healthcare, health information, and living conditions.

In previous studies, the adverse effects of CKD, CVD, diabetes, and multiple diseases on ADLs disability have been widely reported. Plantinga et al. (34) found that the prevalence of disability was 2–3 times higher in people with CKD compared to non-CKD participants, and that older individuals reported higher levels of disability. A study based on the large-scale survey in Shanghai, China (35), indicated that the number of chronic diseases was strongly correlated with BADL and IADL disabilities, with older age, lower income, and female sex being key focus groups, which aligns with our predictions for vulnerable populations. Another CHARLS-based study (36) reached similar conclusions. Regarding cardiovascular system and metabolic risk factors, it was reported that in a study exploring the association between cardiometabolic multimorbidity (CM) and risk of disability (37), participants with CM at baseline had a faster progression of disability compared with those without CM. A study in Taiwan (38) identified cardiovascular disease as an independent factor contributing to elevated disability in people with diabetes. In our study, we did not just focus on single-system diseases, but covered the combined effects between cardiovascular, kidney, and metabolic risk factors. This integrated perspective allows us to gain a deeper understanding of how these systems work together to elevate the risk of ADLs disability.

Most studies of economic factors and ADLs disability are consistent with our conclusion that the risk of ADLs disability is greater in groups with lower economic status. A study exploring the association between multimorbidity and ADLs disability revealed that low-income groups were more likely to have IADL disability (35). Many studies of the association between personal income and ADLs disability have shown a significant association between low income and ADLs disability (39–41). In our study, after including economic status as a covariate in the regression model, there was no significant association with ADLs disability (*p* > 0.05). However, in the study by Fuller-Thomson and Gadalla (42), individual income level was a significant predictor of ADLs disability.

Our research findings provide new leads for the development of targeted policies and interventions to address the increasing burden of CKM syndrome and ADLs disability in China. We suggest that Chinese health authorities take proactive measures to alleviate the staging progression of CKM syndrome to minimize the incidence of ADLs disability. Establish a national surveillance system for CKM syndrome in order to track and assess the staging progression of CKM in real time. Utilize big data and artificial intelligence technologies to identify high-risk populations and implement early interventions. Keep CKM stage at the lowest possible level. Focus should also be placed on strengthening health education and widely promoting knowledge dissemination activities on the prevention and management of CKM syndrome and ADLs disability in the community and schools, so as to enhance the public’s health awareness and self-management ability. A cooperative mechanism involving multiple parties, including community support, health insurance policy makers, medical service providers and pharmaceutical manufacturers, should be established to ensure the accessibility and continuity of comprehensive medical services. Promote a multidisciplinary care model that provides support in various aspects, including nutrition, rehabilitation and psychology. Increase investment in disability research on CKM syndrome and ADLs, provide financial and policy support, and encourage medical institutions and scientific research institutes to carry out relevant research and innovative projects. In this process, special attention should be paid to those who may be seriously affected by CKM Syndrome, including women, the older adults, those with a history of chronic diseases, and those in poorer economic conditions, by designing and implementing special health management programs. This includes regular health checkups, personalized treatment plans, and psychological support services, and improving the distribution of healthcare resources in remote and impoverished areas to ensure that patients in these areas also have access to timely and high-quality healthcare services. In addition, social determinants of health should be taken into account (43), such as social support networks, employment, education, the natural environment and public health, to work toward the realization of health equity.

The study’s strengths include the use of a large nationally representative sample and a longitudinal study design. However, our study has several limitations. First, in order to more accurately characterize CKM stages with data from CHARLS, we had to combine stages 3 and 4 of the original CKM stages, and this combination may have made it difficult to capture subtle differences between the two stages. Besides, information about chronic conditions and ADLs disability was obtained through self-report, introducing the potential for recall bias. In the future, we hope to obtain more suitable data for calculating CKM stages and more scientific methods for characterizing the distribution of CKM syndrome in the Chinese population, to more accurately explore the association between CKM syndrome and ADLs disability.

This study provided evidence that CKM syndrome is a risk factor for ADLs disability. We also found that older age and poorer socioeconomic status increase the risk of ADLs damage. Therefore, it is of significant public health importance for Chinese health authorities to monitor and control the progression of CKM staging in the population, especially among key groups, to reduce the risk of ADLs disability in middle-aged and older adults individuals.

## Data Availability

Publicly available datasets were analyzed in this study. This data can be found here: https://charls.charlsdata.com/users/sign_in/en.html.
